# Prevalence of the *Helicobacter pylori babA2* Gene in Children Mainly Depends on the PCR Primer Set Used

**DOI:** 10.1155/2020/4080248

**Published:** 2020-08-13

**Authors:** Anja Šterbenc, Maja M. Lunar, Matjaž Homan, Boštjan Luzar, Nina Zidar, Mario Poljak

**Affiliations:** ^1^Institute of Microbiology and Immunology, Faculty of Medicine, University of Ljubljana, Zaloška 4, Ljubljana 1000, Slovenia; ^2^Department of Gastroenterology, Hepatology and Nutrition, University Children's Hospital, Faculty of Medicine, University of Ljubljana, Vrazov Trg 2, Ljubljana 1000, Slovenia; ^3^Institute of Pathology, Faculty of Medicine, University of Ljubljana, Korytkova 2, Ljubljana 1000, Slovenia

## Abstract

Various polymerase chain reaction- (PCR-) based methods with varying positivity rates were designed to detect the *Helicobacter pylori babA2* gene. To compare different primer sets, *babA2* prevalence was determined in 279 *H. pylori*-positive pediatric samples using the 832 bp, 139 bp, and 271 bp PCR primer sets, resulting in 34.0%, 51.3%, and 79.6% prevalence of the *babA2* gene, respectively. The *babA2* status determined using the 832 bp and 139 bp PCR primer sets significantly correlated with bacterial density and activity of inflammation, whereas no such correlations were found using the 271 bp PCR primer set. The 139 and 832 bp PCR primer sets concordantly detected the *babA2* gene in 93 cases; however, in comparison to the 832 bp PCR primer set, the 139 bp PCR primer set detected additional 50 *babA2* cases, whereas only two 832 bp positive cases were missed. The 271 bp PCR primer set missed 32 *babA2* cases that were 832 bp and/or 139 bp PCR positive, but tested solely positive in 109 cases. Interestingly, cloning of a subset of 271 bp PCR positive samples revealed amplification of the *babA*/*B* gene chimera. Hence, in our opinion, the 271 bp PCR protocol is not a reliable diagnostic tool for detecting the *babA2* gene in children. Our results reaffirm previous observations that the use of certain *babA2* PCR primer sets can significantly impact estimation of the prevalence and clinical relevance of the *H. pylori babA2* gene in children, suggesting *babA2* detection methods should be carefully selected.

## 1. Introduction

Adherence factors of *Helicobacter pylori* are considered one of the most important virulence factors, which enable long-term persistence of the bacteria in the human stomach [1, 2]. *H. pylori* genome encodes a variety of outer membrane proteins (OMPs), including blood group antigen binding adhesion (BabA) proteins. Three *bab* paralogues have been described thus far: BabA, BabB, and BabC [3]. BabA is one of the most studied *H. pylori* adhesins, capable of binding to Lewis^b^ (Le^b^) and related ABO antigens on gastric epithelial cells [1], which in turn results in increased pathogenicity of *H. pylori* and may play a crucial role in the development of *H. pylori* related gastric pathology such as severe gastritis, peptic ulcers, and gastric adenocarcinoma [4–7]. In contrast, the role of BabB and BabC as adhesins has not been demonstrated yet. The BabA protein is encoded by the *babA2* gene, whereas the *babA1* gene harbors a 10 bp deletion, resulting in protein's inability to interact with Le^b^ antigens [1]. Interestingly, it has been shown that the presence of the *babA2* gene may not uniformly reflect the functional status of the gene [3, 8]. Based on the level of BabA protein production, strains can be divided into BabA high producers (Le^b^ binding activity, contain *babA2* gene), BabA low producers (lacking Le^b^ binding activity but contain *babA2* gene), and BabA nonproducers (lacking Le^b^ binding activity, no detectable *babA2* gene) [8].

*babA* and *babB* exhibit substantial N- and C-terminal identity [1, 9]. Recombination events between *babA* and *babB* appear to be relatively common and have previously been demonstrated for both East Asian and Western strains [10]. Moreover, these intragenomic translocations between the *babA* and *babB* genes are thought to be the main mechanism of BabA expression regulation [3]. Replacement of first 56 bp in the 5′ region of the *babB* with *babA* gene (e.g., *babA/babB* chimera) was first identified in clinical isolates [10] and later also reported during experimental *H. pylori* infection in Rhesus macaques [11]. Somewhat less common are *babB/A* chimera. An analysis of the chimeric *babB/A* gene revealed that the first 47 bp were specific for *babB*, followed by a 66 bp fragment that was shared between both *babA* and *babB*, while the 3′ region showed higher sequence similarity with *babA*. Interestingly, this chimeric BabB/A protein was shown to have the Le^b^ binding capacity [12]. The plasticity of OMPs, not only across different strains but also within a strain colonizing an individual patient, can be regarded as an evolutionary asset, enabling better adaptation of *H. pylori* to the harsh milieu of the human stomach [13].

Various polymerase chain reaction- (PCR-) based methods have been used to detect the *babA2* gene [8]. The majority of studies evaluating the BabA status used PCR primers, designed by Gerhard et al. [4], which aim to detect the 10 bp deletion that distinguishes between the *babA1* and *babA2* genes. These primers target the highly polymorphic zone of the *babA2* gene, amplifying an 832 bp long fragment. Another commonly used PCR primer set was designed by Sheu et al., targeting the promoter region and thus amplifying a 271 bp fragment of the *babA2* gene [14].

In our previous study, we evaluated the *babA2* gene prevalence and clinical relevance in Slovenian children using newly developed primers designed by our research group [15]. Interestingly, while additionally testing a subset of samples with the 832 bp PCR primer set, we noticed considerable differences in the prevalence of the *babA2* gene depending on the type of PCR primer set used [15]. Hence, in order to determine whether the use of a specific PCR primer set influences the *babA2* gene prevalence and clinical relevance in a pediatric population, we performed a head-to-head comparison of *babA2*-specific PCR primer set targeting the 139 bp of the *babA2* gene designed by our research group [15] with two widely used *babA2* PCR primer sets targeting 832 bp [4] and 271 bp segments [14] of the *babA2* gene.

## 2. Materials and Methods

### 2.1. Patients

The study protocol has been described in detail previously [15–17]. Briefly, gastric biopsy samples were obtained from 279 consecutive *H. pylori*-positive children (107 male, 172 female; age range, 5 months–18 years) who had undergone upper endoscopy in the University Children's Hospital in Ljubljana, Slovenia, during the period 1999–2011. Exclusion criteria included previous antibiotic treatment of *H. pylori* infection and usage of nonsteroidal anti-inflammatory drugs, proton pump inhibitors, or H2 receptor blockers within 30 days prior to the endoscopic examination.

### 2.2. Histology

At endoscopy, four biopsy samples were obtained from the gastric antrum and corpus from each patient. Two biopsy samples were used for rapid urease testing (*H. pylori* Quick Test; Biohit Diagnostics, Helsinki, Finland) and the rest were subjected to histopathological examination [16, 17]. According to the updated Sydney classification, two experienced pathologists evaluated the following histological features: density of *H. pylori* colonization, activity of inflammation, chronic inflammation, atrophy, and intestinal metaplasia [18].

### 2.3. *H. pylori* DNA Extraction and *babA2* Genotyping

As described in detail previously [16, 17], *H. pylori* DNA was extracted from the biopsy samples used for the rapid urease testing using the QIAamp DNA Mini kit (Qiagen GmbH, Hilden, Germany). The detection of the *babA2* gene was performed in all *H. pylori* samples using a PCR primer set targeting a 139 bp fragment of the central region of *babA2* that was designed by our research group [15] and two widely used PCRs, targeting 832 bp [4] and 271 bp [14] fragments of a 5′ part of *babA2* (Table 1).

The 139 bp *babA2* PCR protocol was performed using a HotStarTaq Plus DNA Polymerase kit (Qiagen, Hilden, Germany), as described previously [15]. The 832 and 271 bp PCR protocols were performed using AmpliTaq Gold DNA Polymerase kit (Applied Biosystems, Foster City, CA, USA) and FastStart Taq DNA Polymerase kit (Roche, Basel, Switzerland) as described previously [4, 14]. The resulting 139 bp and 832 bp amplicons were analyzed on a 2% agarose gel and only a subset of amplicons (15 PCR products per each PCR protocol) was sequenced with the same primers as those used for initial PCR [19] because both PCR protocols have already been extensively evaluated in previous studies [4, 15].

Sequencing of 13 randomly selected solely 271 bp PCR positive samples and two samples that tested positive using all three PCRs was attempted; however, we were not able to obtain the respective sequences due to technical difficulties. Hence, these samples were purified using QIAquick PCR Purification kit (Qiagen) and subsequently cloned, using CloneJET PCR Cloning Kit (Thermo Fisher Scientific, Vilnius, Lithuania). A sticky-end cloning protocol was used and the overnight transformation was performed using One Shot® TOP10 Chemically Competent *E. coli* (Invitrogen, Carlsbad, USA), according to the manufacturer's instructions. Recombinant plasmid clones from the transformed bacterial culture were verified for the corresponding *H. pylori* inserts with colony PCR and sequencing of PCR products performed using pJET1.2 sequencing primers (pJET1.2 F 5′-CGACTCACTATAGGGAGAGCGGC-3′ and pJET1.2 R 5′-AAGAACATCGATTTTCCATGGCAG-3′), according to the manufacturer's instructions.

The obtained sequences were annotated by BLAST nucleotide search against the current NCBI database. The sequences were aligned and edited using BioEdit ClustalW multiple sequence alignment together with *babA2* and *babB* reference sequences obtained from publically available NCBI database. Phylogenetic analysis was performed using PhyML (v3.0) integrated in the Phylogeny.fr platform, including HKY85 as a substitution model with 4 gamma categories. The proportion of invariable sites and gamma shape parameter were estimated directly from the data. The obtained phylogenetic tree was graphically presented with Figtree version 1.4.3 (http://tree.bio.ed.ac.uk/software/figtree/) and phylogenetic relationships were assessed according to the approximate likelihood ratio test (aLRT) branch support values [20].

In addition, because some of the sequences obtained exhibited mismatch with the *babA2* reference sequences, amplification of *babA/B* chimera was suspected. The presence of chimeric *babA/B* gene was studied by performing bootscanning method available in Simplot, version 3.5.1., with 500 bootstrap replicates on a varying window sizes (120–60 bp) with a 10 bp sliding window [21]. Two separate slices of alignments were generated and phylogenetic analysis was performed, as described above.

### 2.4. Statistical Analysis

The data were analyzed using the SPSS 11.0 statistical package (SPSS GmbH Software, Munich, Germany). Statistical significance of differences between evaluated PCR primer sets was evaluated using the *t*-test and Chi square test with a *p* value of less than 0.05 considered as statistically significant.

## 3. Results

### 3.1. Prevalence of the *babA2* Gene

Using 832 bp [4], 139 bp [15], and 271 bp [14] PCR primer sets, *babA2* was detected in 34.0% (95/279), 51.3% (143/279), and 79.6% (222/279) of *H. pylori* samples studied, respectively. Concordant results (either all positive or negative for *babA2*) were obtained in 35.5% (99/279).

**Figure 1:**
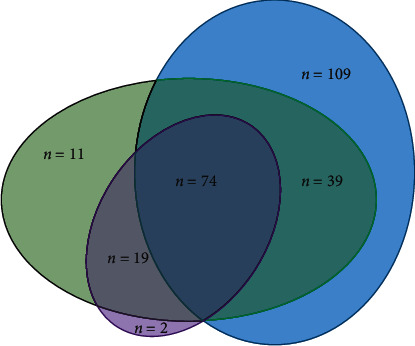
Venn diagram showing all possible combinations of *babA2* positive strains determined by the 832 bp (purple), 139 bp (green), and 271 bp (blue) *babA2* PCR protocols.

As shown in Figure 1, the 139 bp and 832 bp *babA2* PCR primer sets concordantly detected *babA2* in 93 cases; however, in comparison to the 832 bp PCR primer set, the 139 bp PCR primer set additionally detected 50 *babA2*-positive samples while missing two samples that were babA2 positive according to the 832 bp PCR primer set. In total, 109 samples were concordantly negative using the 832 bp and 139 bp PCR primer sets but tested positive using the 271 bp PCR primer set. Furthermore, the 271 bp PCR primer set missed 32 *H. pylori* samples that tested positive using the 139 bp and/or 832 bp PCR primer sets.

### 3.2. Association of *babA2* Genotypes with Histological Parameters in Gastric Mucosa

In order to evaluate to what extent a certain *babA2* PCR protocol affects result interpretation on the clinical relevance of the *babA2* gene, we assessed potential correlations between the presence of the *babA2* gene detected by three different PCR protocols and five histological parameters. As shown in Table 2, *H. pylori* strains determined as *babA2* positive by the 139 bp PCR protocol were strongly associated with two out of five histological parameters studied: *H. pylori* density score (*p*=0.007) and activity of inflammation (*p*=0.003), while the association with chronic inflammation of the gastric mucosa was borderline (*p*=0.063). Similarly, when using the 832 bp PCR primer set, a strong association was observed between the *H. pylori* strains determined as *babA2* positive and *H. pylori* density score (*p*=0.015) and activity of inflammation (*p*=0.016) (Table 2). In contrast, *babA2* positive strains confirmed by the 271 bp PCR showed no statistically significant associations with any of the five histological parameters evaluated (Table 2).

### 3.3. Amplicon Sequencing, Cloning, and Phylogeny

**Figure 2:**
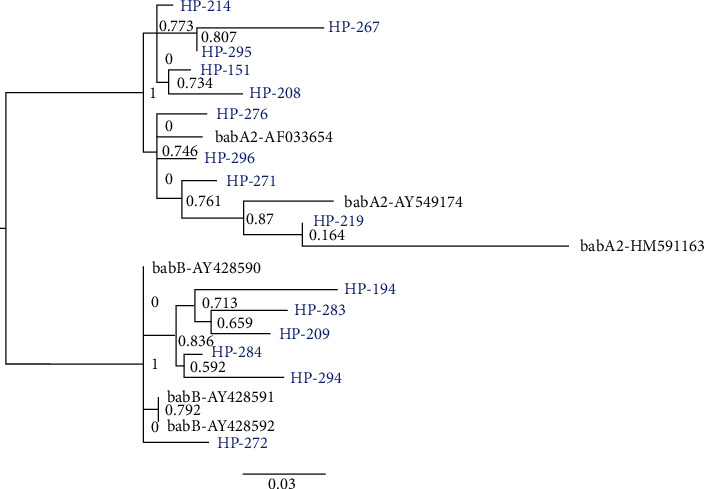
Phylogenetic tree showing three *babA2* and three *babB* gene reference sequences and 15 strains, which tested positive with the 271 bp PCR primer set [14]. Numbers at nodes show aLRT branch support values. *H. pylori* isolates are named as HP-consecutive number (blue), the reference *babA2* and *babB* gene sequences, obtained from publically available NCBI database, are provided with the respective accession numbers (black).

Sanger sequencing of a subset (15 cases each) of randomly selected 139 bp and 832 bp amplicons confirmed the presence of *babA2*-specific sequences. However, when using the 271 bp PCR primer set, the amplicons showed substantial diversity in amplicon length, ranging from approximately 240 bp to 300 bp. Direct sequencing of these variable PCR amplicons was attempted; however, we were unable to obtain complete DNA sequences, most likely due to the presence of repetitive elements. Hence, cloning of 15 randomly selected 271 bp PCR positive samples with amplicon sizes not corresponding to 271 bp was performed. The following cases were evaluated: 13 samples that were solely positive using the 271 bp PCR (samples HP-151, HP-194, HP-208, HP-209, HP-214, HP-267, HP-271, HP-272, HP-276, HP-283, HP-294, HP-295, and HP-296), and two samples that were positive using all three PCR protocols (samples HP-219 and HP-284). The phylogenetic analysis (Figure 2) of the obtained sequences showed clustering into two distinct groups with significant aLRT branch support values of more than 0.99. Namely, 9/15 sequences clustered together with the *babA2* gene reference sequences, while 6/15 sequences clustered with the *babB* gene references.

**Figure 3:**
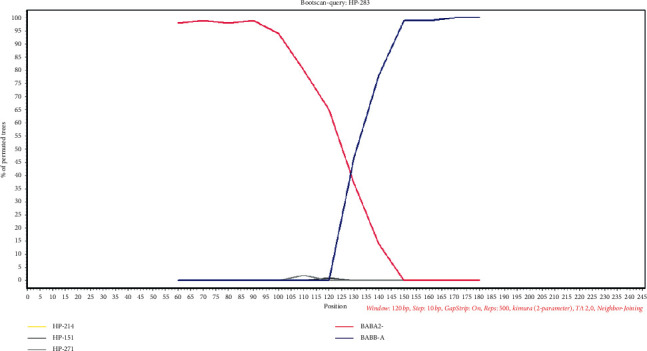
Analysis of the *babA* and *babB* gene recombination. Bootscan of sample HP-283 is presented using Simplot with 500 bootstrap replicates, window size of 120 bp and 10 bp sliding window.

**Figure 4:**
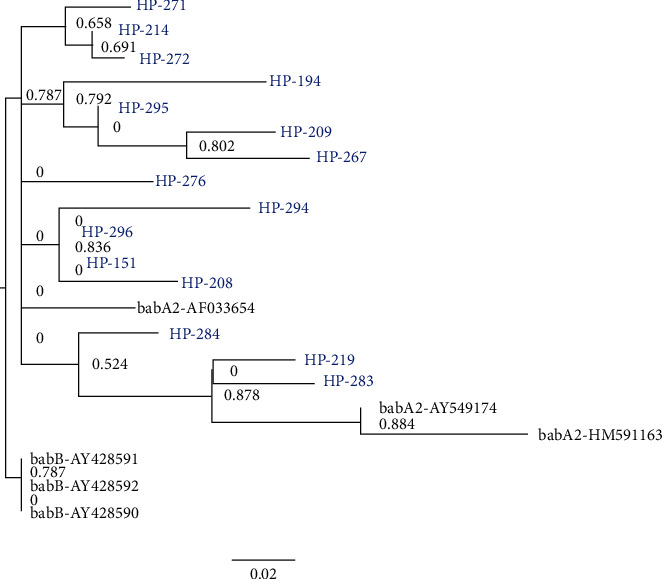
Phylogenetic tree of the first section (1–156 bp) of the alignment showing phylogenetic clustering of 15 generated sequences with the *babA2* reference sequences. *H. pylori* isolates are named as HP-consecutive number (blue), the reference *babA2* and *babB* gene sequences, obtained from publically available NCBI database, are provided with the respective accession numbers (black).

Simplot analysis indicated possible evidence of recombination (Figure 3), and therefore separate phylogenetic analyses were performed for two slices of the alignment (1–156 bp and 157–246 bp), according to the average recombination breakpoint of analyzed samples. The obtained phylogenetic tree of the first section of the alignment displayed phylogenetic clustering of all 15 generated sequences with the *babA2* reference sequences, although with less significant aLRT value (0.787) (Figure 4). On the other hand, the second part of the alignment displayed the same phylogenetic relationship, as did the previous phylogenetic analysis performed on the complete alignment (Figure 2). Therefore, our results indicate that the 271 bp primers set amplified chimeric *babA/B* gene.

## 4. Discussion

In this study, we performed a head-to-head comparison of *babA2* gene-specific PCR primer set designed by our research group [15] with two previously established *babA2* PCR protocols in order to assess the *babA2* prevalence using the largest collection of *H. pylori* positive biopsies obtained from children (*n* = 279). Interestingly, the prevalence of the *babA2* gene in our study varied significantly according to the PCR primer set used; the *babA2* positivity rate using the 271 bp PCR primer set was more than double of that obtained by the 832 bp PCR primer set (79.6% versus 34.0%), whereas the 139 bp PCR primer set yielded intermediate *babA2* positivity rates (51.3%). In majority of previous studies in adults and children [4, 6, 22–30], the *babA2* status was determined using the 832 bp PCR primer set, originally designed by Gerhard et al. [4]. According to the literature data, the prevalence of the *babA2* gene in adults determined by using these primers is highly variable, ranging from 32 to 72% in Western countries and from 64 to 85% in Asian countries [8], while in children, the *babA2* gene positivity ranges from 17 to 84% [22–30]. Sheu et al. [14] designed a second widely used PCR primer set, which amplifies a 271 bp fragment of the *babA2* gene. In comparison to the 832 bp PCR primer set, the *babA2* prevalence determined using the 271 bp PCR primer set in adults was generally higher, ranging from 41 to 100% [7, 14, 31, 32]. To the best of our knowledge, only one previous study used the 271 bp PCR primer set for detecting the *babA2* gene in children, yielding a 41% prevalence among Turkish pediatric population [33].

Adherence of *H. pylori* to gastric epithelial cells is one of the most important contributing factors to the pathogenicity of the bacteria [34]. The *babA2* gene encodes a protein that enables *H. pylori* attachment to Le^b^ epitopes on human epithelial cells and thus the delivery of bacterial virulence factors into host target cells, resulting in gastric tissue damage [35]. In this study, we also aimed to evaluate whether the use of certain *babA2* PCR primer set influences the clinical relevance of the *babA2* gene. Interestingly, we found a strong correlation between *babA2* positive status and the density of *H. pylori* colonization, as well as the degree of active inflammation determined by the 139 and 832 bp PCR primer sets (Table 2). However, no significant associations were found between the *babA2* status and *H. pylori* density score, degree of activity, or chronic inflammation of the gastric mucosa when using the 271 bp PCR primer sets. In this study, *babA2* positive strains (determined with any of the three PCR methods used) were not found more frequently in patients with gastric atrophy or intestinal metaplasia, suggesting involvement of other virulence or host factors in the disease progression. Nevertheless, data regarding the *babA2* gene prevalence and clinical significance should be interpreted with caution, since the use of a particular *babA2* PCR primer set results in significant differences in the *babA2* gene detection rates. Moreover, conflicting results on the association between the presence of the *babA2* gene and gastric cancer obtained in previous studies could be due to using only a single PCR primer set for detection of the *babA2* gene, an approach that may not reflect the actual status of the BabA protein expression [35]. Chang et al. [36] even proposed using multiple pairs of PCR primers to provide a more reliable estimation of the BabA status and its influence on the risk of gastric cancer, especially in isolates from East Asia with nearly 100% prevalence of the *babA2* gene [14, 36].

The *babA* gene was initially cloned from *H. pylori* strain CCUG17875, which contains a silent (nonfunctional) *babA1* gene and an expressed *babA2* gene. The sequences of these two genes differ only by the presence of a 10 bp deletion in the signal peptide sequence of *babA1*, which eliminates its translational initiation codon. However, later analysis showed that naturally occurring *babA1* sequences are very rare, suggesting PCR-based methods designed to detect the 10 bp deletion (e.g., the 832 bp PCR primer set) do not reliably reflect the BabA status [8, 13]. As shown in Figure 1, the 139 bp PCR primer set detected 50 additional *babA2* positive cases compared to the widely used 832 bp PCR primer set, suggesting superior sensitivity of the 139 bp PCR primer set for detecting the *babA2* gene. Thus, our data support previous observations of ineffectiveness and underestimation of the *babA2* gene prevalence when using the 832 bp PCR primer set [6, 8, 22]. Although the forward babA2F primer is located within a relatively conserved 5′ region, the presence of single nucleotide polymorphisms may nonetheless impede effective amplification. Moreover, because the reverse babA2R primer was designed to anneal within a highly variable region in order to reliably discriminate between various *bab* homologues, this may invariably lead to underestimation of the *babA2* prevalence [6]. According to Fujimoto et al. [8], the sensitivity of the *babA2* PCR primer sets that were designed to discriminate between the *babA2* and *babA1* genes was shown to be only approximately 36–49%, thus missing a significant proportion of the BabA high and low producers. Nevertheless, significantly higher prevalence of the *babA2* gene (e.g., >70%) obtained from Asian isolates using the same primer set highlights the significance of geographical variability among *H. pylori* strains [6, 8, 22].

The issue of nucleotide mismatching in the primer binding region of the 832 bp PCR primer set was circumvented when designing primers for the 139 bp PCR protocol by systematic investigation of the *babA2* genomic diversity of geographically distinct *H. pylori* samples and the consequent addition of more *babA2* specific primers to cover all variations of the *babA2* gene known at the time of primer design. In specific, three forward and one reverse primer were designed on the basis of multiple alignments of 94 *babA2* and 24 *babB* sequences [15]. To avoid amplifying nearly identical 5′ and 3′ regions of the *babA* and its paralogue *babB*, a set of three forward and one reverse primer was selected in the central region of the *babA2* gene which target conserved regions of the *babA2*, yielding a 139 bp *babA2* gene-specific amplicon. However, despite our best efforts to design a PCR primer set that would amplify all *babA2* gene variants, the 139 bp PCR primer set failed to detect two *babA2* positive cases determined by the 832 bp PCR primer set, suggesting addition of more primers or use of a combined approach (e.g., concurrent amplification with the 139 and 832 bp PCR primer sets) may be used in order to increase the sensitivity of the PCR. Nevertheless, as shown by Pride et al. [37], sequencing of the *babA* and *babB* genes from *H. pylori* strains from around the world showed high sequence variability in different strains, thus making it difficult to construct a universally applicable primer set.

The 271 bp PCR primer set uses a forward bab7-F primer that is located in the promoter region of the *babA2* gene in combination with a unique reverse primer from the 5′ region of the *babA2* gene. A study by Fujimoto et al. [8] showed that the 271 bp PCR primer set originally developed by Sheu et al. [14] is not a reliable molecular tool for detecting the *babA2* gene in adults, because it frequently amplifies also the *babB* gene sequences and, consequently, produces *babA2* false-positive results. Interestingly, the reverse bab7-R primer sequence matches exactly both the *babA2* and *babB* gene sequences, which may be the reason for the occasional nonspecific *babB* gene amplification. Our study showed that the inadequacy of the 271 bp PCR for detecting the *babA2* gene also exists in pediatric population. In this study, Sheu's primers generated not only the predicted 271 bp amplicons, but also amplicons of various sizes, ranging from approximately 240 to 300 bp, which was already noted previously [32]. Unlike the study by Mattar et al. [32], in which the two randomly sequenced PCR amplicons were *babA2* in origin, 6/15 cloned sequences from our study were shown to belong to the *babB* cluster, whereas the rest of the sequences showed higher similarity to the *babA2* gene sequences. As shown in Figure 2, significant aLRT branch support values were observed, thus clearly demonstrating clustering of the strains into two distinct groups: the *babA2* and *babB* gene group. To our surprise, sequencing of one out of two cloned 271 bp amplicons that tested positive using all three PCR protocols yielded *babB*-like sequences. Indeed, further analysis revealed that the 271 bp PCR primer set in fact detected *babA/B* gene chimeras (Figure 3), which appear to be relatively common among bacterial isolates [38]. It has previously been shown that, in isolates lacking the *babA* gene (11/34; 33%), the chimeric *babA/B* gene which results in loss of Le^b^ activity due to inhibition of the BabA expression predominates chimeric *babB/A* gene that otherwise subjects protein expression to phase variation [38]. Furthermore, 32 *H. pylori* samples that were positive with the 139 bp and/or 832 bp PCR primer sets tested negative with the 271 bp PCR primer set, indicating that this PCR protocol may also generate *babA2* false-negative results (Figure 1). This may be due to significant variations in *babA2* gene sequences among the isolates.

To the best of our knowledge, no PCR-based method can be considered as “gold standard” for detecting the *babA2* gene. Moreover, PCR-based determination of the functional status of the gene is importantly hampered by several factors, namely varying sensitivity and specificity of different *babA2* PCR primer sets, also reflecting a high degree of heterogeneity among different *H. pylori* isolates, as well as the presence of BabA low producers: *H. pylori* strains that in spite of detectable *babA2* gene lack Le^b^ binding activity [3, 8]. Despite being relatively inexpensive and, most importantly, easy to perform and to interpret, PCR-based methods will probably be soon replaced by whole-genome sequencing. It has already been shown that whole-genome sequencing can be used for precise and fast characterization of *H. pylori* virulence genes, including *babA2* [39].

There are some limitations to this study, including the relatively low number of sequenced 271 bp PCR amplicons. In addition, determining which *bab* gene occupies which genomic locus would also provide additional information regarding the usefulness of respective *babA2* PCR primer sets. Because PCRs were performed using tissue biopsies and not *H. pylori* isolates, it is likely that *H. pylori* subpopulations were not adequately evaluated. Hence, future studies evaluating these PCR primer sets should be performed on multiple *H. pylori* isolates obtained from various gastric biopsies.

## 5. Conclusions

This study showed that the estimation of the prevalence and clinical relevance of the *H. pylori babA2* gene in children mainly depends on the PCR primer set used. The 139 bp PCR primer set exhibited superior sensitivity than the previously most frequently used PCR primer set targeting 832 bp fragment of the *babA2* gene. Similar to the 832 bp PCR primer set, the *babA2* status determined by the 139 bp PCR primer set statistically significantly correlated with two out of five histological parameters evaluated, whereas such correlation was not evident when the 271 bp PCR primer set was used. Comparison of three different *babA2* PCR primer sets on the largest number of pediatric *H. pylori* samples to date confirmed previous observations that the 271 bp PCR protocol is a not reliable diagnostic tool for the detection of the *babA2* gene and should thus be avoided.

## Figures and Tables

**Table 1:** PCR primers used for the *babA2* gene detection.

Primer	Sequence (5′-3′)	Size (bp)	Reference
babA2F	AATCCAAAAAGGAGAAAAAGTATGAAA (s)	832	Gerhard et al. [4]
babA2R	TGTTAGTGATTTCGGTGTAGGACA (as)		

bab7-F	CCAAACGAAACAAAAAGCGT (s)	271	Sheu et al. [14]
bab7-R	GCTTGTGTAAAAGCCGTCGT (as)		

babA (B)-F1	TATCAAGCCGTGCTTTT (s)	139	Homan et al. [15]
babA (B)-F2	TATCAGGCCGTGCTTTT (s)		
babA (B)-F3	TATCAAGCGGTGCTTTT (s)		
babA2k-rew2	CAACGAGCCAGGGTATC (as)		

s: sense; as: antisense.

**Table 2:** Association between the presence of the *babA2* gene determined by 823 bp, 139 bp, and 271 bp PCR primer sets and histological parameters.

	Number of specimen				Total	Mean score	*p* value
	Histological score						
	0	1	2	3			
	Bacterial density						
*832 bp PCR primer set* ^*a*^							
Positive	1	3	31	60	95	2.58	0.015
Negative	0	13	87	84	184	2.39	
*139 bp PCR primer set* ^*b*^							
Positive	1	5	50	87	143	2.56	0.007
Negative	0	11	68	57	136	2.34	
*271 bp PCR primer set* ^*c*^							
Positive	1	12	95	114	222	2.45	0.910
Negative	0	4	23	30	57	2.46	

Activity of inflammation							
*832 bp PCR primer set* ^*a*^							
Positive	3	39	43	10	95	1.63	0.016
Negative	8	104	66	6	184	1.38	
*139 bp PCR primer set* ^*b*^							
Positive	3	63	64	13	143	1.61	0.003
Negative	8	80	45	3	136	1.32	
*271 bp PCR primer set* ^*c*^							
Positive	7	116	88	11	222	1.46	0.366
Negative	4	27	21	5	57	1.47	

Chronic inflammation							
*832 bp PCR primer set* ^*a*^							
Positive	0	7	76	12	95	2.05	0.902
Negative	1	15	145	23	184	2.03	
*139 bp PCR primer set* ^*b*^							
Positive	0	8	111	24	143	2.11	0.063
Negative	1	14	110	11	136	1.96	
*271 bp PCR primer set* ^*c*^							
Positive	1	17	175	29	222	2.05	0.901
Negative	0	5	46	6	57	2.02	

	Atrophic changes						
*832 bp PCR primer set* ^*a*^							
Positive	68	27	0	0	95	0.28	0.346
Negative	137	44	3	0	184	0.27	
*139 bp PCR primer set* ^*b*^							
Positive	104	38	1	0	143	0.28	0.758
Negative	101	33	2	0	136	0.27	
*271 bp PCR primer set* ^*c*^							
Positive	158	61	3	0	222	0.30	0.191
Negative	47	10	0	0	57	0.18	

Intestinal metaplasia							
*832 bp PCR primer set* ^*a*^							
Positive	90	5	0	0	95	0.05	0.569
Negative	177	7	0	0	184	0.04	
*139 bp PCR primer set* ^*b*^							
Positive	138	5	0	0	143	0.03	0.497
Negative	129	7	0	0	136	0.05	
*271 bp PCR primer set* ^*c*^							
Positive	214	8	0	0	222	0.04	0.257
Negative	53	4	0	0	57	0.07	

^*a*^Gerhard et al. [4]; ^*b*^Homan et al. [15]; ^*c*^Sheu et al. [14].

## Data Availability

The data used to support the findings of this study are included within the article.

## Abbreviations

PCR: Polymerase chain reaction
BabA: Blood group antigen binding adhesion
Le^b^: Lewis^b^
aLRT: Approximate likelihood ratio test
bp: Base pairs.
